# Diarrheal correlates associated with enteric bacterial infections among children below five years in Murang'a County, Kenya

**DOI:** 10.11604/pamj.2019.34.170.17403

**Published:** 2019-12-02

**Authors:** Oliver Waithaka Mbuthia

**Affiliations:** 1 Department of Medical Laboratory Science, School of Medicine, Kenyatta University, Nairobi, Kenya

**Keywords:** Diarrhea, correlates, sanitation, hygiene, demographics, bacteria, *enterobacteriaceae*, etiology, virulence

## Abstract

**Introduction:**

The burden of childhood diarrheal disease has resulted in massive mortality and morbidity globally. Children below 5 years in sub-Saharan Africa are most implicated by diarrheal illnesses resulting in numerous medical consultations, admissions, and deaths despite the disease being easy to prevent and control. The study aimed to determine the correlates of enteric bacterial infection causing diarrhea.

**Methods:**

During the months of April-October 2017, 163 children below five years presenting with diarrhea were randomly selected in Murang'a and Muriranja's hospitals. Bacterial agents were identified and correlates of diarrhea determined. The study used a hospital-based cross-sectional study design. A standardized questionnaire was used to collect information from the guardian. Statistical analyses were performed using STATA v. 13.

**Results:**

Forty-nine children were infected with enteric pathogenic bacteria (Enterotoxigenic *Escherichia coli*, Enteropathogenic *Escherichia coli*, Enteroaggregative *Escherichia coli*, *Salmonella*, *Shigella*, and Vibrio species). Factors associated with infection by these bacteria among the 49 children were evaluated. Children between 0-12 months (OR 0.3, 95% CI 0.1-0.8), those fed exclusively on breast milk (OR 0.3, 95% CI 0.09-0.9) and children weighing 1-5 kilograms (OR 0.2, 95% CI 0.04-0.9) were less likely to be infected with these enteric pathogenic bacteria. Female participants (OR 1.8, 95% CI 1.1-3.4) were nearly twice likely to be infected with enteric pathogenic bacteria. Children who presented with watery stool (OR 0.4, 95% CI 0.2-0.9) or mucoid stool (OR 0.3, 95% CI 0.2-0.7) remained associated with enteric pathogenic bacterial infection but less likely to be infected compared to those who presented with watery-blood stained stools. Piped water (OR 0.01, 95% CI 0.01-0.4) was less associated with enteric bacterial infection than water stored in jerry-cans while storing water without a lid (OR 1.9, 95% CI 1.1-3.7) was more likely to lead to bacterial infection. Hand washing after toilet use (OR 1.6, 95% CI 1.1-2.7) was associated with enteric bacterial infection compared to hand washing before meal preparation.

**Conclusion:**

Sanitation, hygiene, nutritional and clinical factors were associated with enteric bacterial infections causing diarrhea among children below five years in the study area. Childhood diarrhea in Murang'a County is a major public health problem.

## Introduction

Tremendous efforts, through global initiative, has seen diarrheal mortalities reduce substantially in the last two decades but diarrhea remains the top two killer disease, responsible for 72% deaths of children below two years [1], claims the lives of about 800,000 children below five years annually [2], and the leading cause of death among children of this age in Sub-Saharan Africa (SSA) [3]. Fischer Walker *et al.* [1] in the year 2011 observed Kenya as among the ten countries in SSA where childhood mortality associated with diarrhea disease burden is alarming. Sixteen percent (16%) of deaths in Kenya have been associated with diarrhea among children who do not live to see their 5th birthday [4]. The burden of diarrhea in Kenya remains elusive with unacceptable morbidity and mortalities among children below five years. Deficiencies in preventive, diagnostic and treatment infrastructures are still major stabling blocks both at local and community levels [5]. Preventable diseases account for 80% medical consultations in Kenya while half of these are sanitation and hygiene-related such as diarrhea which is ranked as top three illness among children below 5 years [6].

Transmission of enteric bacteria-causing diarrhea is mainly through the fecal-oral route. Simple interventions that have proven feasible in the prevention of diarrheal illnesses [7, 8], but certain enteric bacteria continue to proliferate in nearly all environments and causes diarrhea among children below 5 years. Hygiene, sanitation, nutrition, and socioeconomic factors are key and a priority against transmission and infection of enteric bacterial pathogens causing gastroenteritis [9]. Cheap, efficient and sustainable interventions such as water treatment [10], hand washing with soap and water [11], and balanced diet have been demonstrated to effectively minimize diarrheal disease. Educating the community on the importance of behavioral changes through health promotion programs offers a long-term solution in the fight against enteric diarrheal illnesses among children below five years. The study identified factors associated with enteric bacterial infection among children below 5 years calling the need for immediate action for implementation of public health interventions.

## Methods

**Study site:** the study was carried out in Murang'a County, Kenya, located about 80 kilometers from the Kenyan capital, Nairobi. Two major referral hospitals within the county were selected (Murang'a referral hospital and Muriranja's tier 4 hospital).

**Research design:** a hospital-based cross-sectional study approach was used.

**Target population:** the research assessed children below five years who sought healthcare due to diarrheal illness within Murang'a County Referral Hospital and Muriranja's tier 4 Hospital. Written informed consent was obtained from the child's caretaker and upon signing questions about their child/(ren) were directed to them.

**Inclusion criteria:** children below five years who reported with a loose stool at Murang'a Referral Hospital and Muriranja's tier 4 Hospital, were residents of Murang'a county and caretakers of those who gave consent to participate in the study.

**Sampling design:** sample selection was done using the systematic random sampling where the first unit (case) was selected randomly in each hospital. The nth case after the starting point followed a systematic selection. The nth case represents the sampling interval which was calculated by dividing the approximate total number of diarrhea cases by the sample size of 163 per facility. Therefore, every 4th case of diarrhea (Muriranja's hospital) and 5th (Murang'a Hospital) ware selected until a sample size of 163 was reached from both hospitals.

**Sample size determination:** applying the formula for estimating the population proportion with specified relative precision described by Daniel [12] setting the α at 0.05, and a detection rate of 12.1% for children below five years infected with diarrheal disease in Murang'a County [13], a total of 163 children were recruited to achieve 0.95 power.

**Data collection instruments:** the procedure that was used in data collection included structured data collection instruments (weighing balance, thermometers and other calibrated equipment). A structured questionnaire containing three sections was directed to the caretaker and question regarding socio-demographic (age, sex, guardianship, education, family type, household population) clinical (weight, nutrition, stool appearance, dehydration, fever, previous history, feeding) and environmental factors (sanitation and hygiene) were asked. Further clinical information was abstracted from the child's clinical card and Mother-Child Health (MCH) booklet.

**Validity:** pre-testing was conducted in the two hospitals prior to validate the research methods and tools.

**Sample and data collection:** registered clinicians physically and clinically examined the child's general health status and recorded. Research assistants guided the child's guardian on the questions contained in the questionnaire and ensured that a stool sample was correctly obtained. Diarrheal stool samples were collected on the day of presentation at the Hospitals using well labeled sterile leak-proof poly-pots. Stool appearance was recorded in the hospital-laboratory on the study questionnaire entries that matched the specimen identification number and then cultured in Cary Blair medium (Oxoid, United Kingdom) which was then properly sealed, labeled and stored at 4-8 ºC for one day. Samples were disposed of as per the standard operating procedure of infectious material. On the 2nd day, the cultured transport media was put in a cool box with frozen ice packs and shipped within 3 hours to the Kenya Medical Research Institute (KEMRI), Center for Microbiology Research in Nairobi. All stool samples were immediately cultured using appropriate media. All bacterial isolates were morphologically and biochemically identified. *Escherichia coli* pathotypes were identified using multiplex Polymerase Chain Reaction (PCR).

**Data analysis and presentation:** frequency (%), mean, standard deviation, and medium (interquartile ranges at 25% and 27%) were used to describe the qualitative and laboratory parameters. Chi-square or Fisher's exact test was used to test for significance where applicable. In bivariate analyses, odds ratios (OR) and 95% confidence intervals (CI) for the association between enteric pathogenic bacterial isolates etiological agent of child diarrhea and socio-demographic, hygienic and environmental characteristics were calculated using Poisson regression. In multivariate analyses, a manual backward elimination approach was used to reach the most parsimonious model, including factors that were associated with infection with enteric pathogenic bacterial isolates at the significance level of P<0.05. All statistical analyses were performed using STATA v 13 (StataCorp LP, College Station, TX, USA).

**Ethical consideration:** ethical approval was granted by Kenyatta University Ethics and Research Review Committee (KU-ERRC) KU/ERC/APPROVAL/VOL.1 (31). Research permit was given by the National Commission for Science Technology and Innovation (NACOSTI) NACOSTI/P/17/15949/16819. Permission was also given by the County Commissioner, director of Health and director of Education. The study was performed in accordance with the Helsinki Declaration [14].

## Results

**Factors associated with diarrheal disease among participants:** the following bacteria have been frequently associated with diarrheal cases in children; Enterotoxigenic *Escherichia coli* (ETEC), Enteropathogenic *Escherichia coli* (EPEC), Enteroaggregative *Escherichia coli* (EAEC), *Salmonella* species, *Shigella* species, and Vibrio species. In this study, there were forty-nine (49) children infected with these bacteria. Factors associated with infection by these bacterial among the 49 children were evaluated.

**Socio-demographic factors:** Table 1 summarizes the socio-demographic factors associated with infection with enteric pathogenic bacteria isolated from children in the study. In the bivariate analysis, participants who were of female (OR 1.8, 95% CI 1.1-3.4) were more likely to be infected with these enteric pathogenic bacteria. Children who were aged between 0 to 12 months (OR 0.3, 95% CI 0.1-0.8) were less to be infected with enteric pathogenic bacteria compared to children aged between 49 to 60 months.

**Table 1:** Socio-demographic factors associated with enteric pathogenic bacterial infection

		Enteric pathogenic bacterial infection			
Variables	Total			P - value	Bivariate
		No	%		OR (95% CI)
**Child gender**					
***Female***	***86***	***33***	***38.4***	***0.044***	***1.8(1.1 - 3.4)***
Male	77	16	20.8	Referent	Referent
**Age (Months)**					
***0-12 months***	***58***	***10***	***17.2***	***0.021***	***0.3(0.1 - 0.8)***
13-24 months	48	17.0	35.4	0.351	0.6(0.3-1.6)
25-36 months	31	12	38.7	0.488	0.7(0.3-1.8)
37-48 months	13	3	23.1	0.22	0.4(0.1-1.7)
49-60 months	13	7.0	53.8	Referent	Referent

No - Number; % - Percentage; OR - Odds ratio; CI - confidence interval

**Feeding and gastrointestinal factors:** among the feeding and gastrointestinal associated factors, in the bivariate analysis, breastfeeding was found associated with enteric pathogenic bacterial infection. Children who were fed exclusively on breast milk (OR 0.3, 95% CI 0.09-0.9) were less likely to be infected with these enteric pathogenic bacteria compared to those who were currently not breastfeeding (Table 2).

**Table 2:** Breastfeeding factors associated with enteric pathogenic bacterial infection

Variables	Total	Enteric pathogenic bacterial infection		P - value	Bivariate
		No	%		OR (95% CI)
**Child breast feeding**					
Yes	84	21	25	0.227	0.7(0.4-1.2)
No	79	28	35.4	Referent	Referent
**Breastfeeding type**					
***Exclusively***	***28***	***3***	***10.7***	***0.047***	***0.3(0.09-0.9)***
Complementary	57	18	31.6	0.671	0.8(0.5-1.6)
Not applicable	78	21	24.7	Referent	Referent
**Age at child stopping breastfeeding**					
≤ 18 months (or 1 year 6 months)	52	18	34.6	0.392	1.3(0.7-2.4)
≥ 19 months (or 1 year 7 months)	24	8	33.3	0.572	1.2(0.6-2.8)
Not application	87	23	26.4	Referent	Referent

No - Number; % - Percentage; OR - Odds ratio; CI - confidence interval

**Child's clinical presentations:** Table 3 summarizes the clinical presentation factors associated with infection with enteric pathogenic bacteria isolated from children in the study. In the bivariate analysis, children who weighed between 1 to 5 kilograms (OR 0.2, 95% CI 0.04-0.9) were less likely to be infected with these enteric pathogenic bacteria compared to those who weighed 16 to 20 kilograms. Further, children who presented with watery stool (OR 0.4, 95% CI 0.2-0.9) or mucoid stool (OR 0.3, 95% CI 0.2-0.7) were less likely to be infected with enteric pathogenic bacteria compared to children who presented with bloody and watery stool.

**Table 3:** Clinical presentation factors associated with enteric pathogenic bacterial infection

		Enteric pathogenic bacterial infection			
Variables	Total			P - value	Bivariate
		No	%		OR (95% CI)
**Fever**					
Yes	153	46.0	30.1	0.295	1.5(0.7-3.4)
No	10	3.0	30	Referent	Referent
**Weight (Kilograms)**					
***1 to 5***	***53***	***11***	***20.8***	***0.041***	***0.2(0.04-0.9)***
6 to10	79	27	34.2	0.143	0.3(0.08-1.4)
10 to15	29	9	31	0.134	0.3(0.06-1.4)
16 to 20	2	2	100	Referent	Referent
**Stool appearance**					
***Watery***	***74***	***21***	***28.4***	***0.018***	***0.4(0.2-0.9)***
***Mucoid***	***69***	***16***	***23.2***	***0.006***	***0.3(0.2 - 0.7)***
Bloody	2	0	0	ND	ND
Bloody and Watery	18	12	66.7	Referent	Referent

No - Number; % - Percentage; OR - Odds ratio; CI - confidence interval; ND - Not done

**Hygiene and sanitation factors:** Table 4 summarizes the hygienic and sanitation related-practices associated with infection with enteric pathogenic bacteria isolated from children in the study. In the bivariate analysis, the type of water source was found associated with enteric pathogenic bacterial infection. Those who sourced water by piping (OR 0.01, 95% CI 0.01-0.4) were less likely to be infected with these enteric pathogenic bacteria (ETEC, EPEC, EAEC, *Shigella*, *Salmonella*, Vibrio cholera) than those who sourced water from water vendors. Storing water without a lid (OR 1.9, 95% CI 1.1-3.7) was associated with enteric pathogenic bacterial infection compared to those who stored water using jerry cans. Washing hands after toilet use (OR 1.6, 95% CI 1.1-2.7) was likely to lead to infection with enteric pathogenic bacteria compared to those who washed hands before meal preparation.

**Table 4:** Hygienic and sanitation factors associated with enteric pathogenic bacterial infection

Variables	Total	Enteric pathogenic bacterial infection		P - value	Bivariate
		No	%		OR (95% CI)
**Source of drinking water**					
***Piped***	***43***	***2***	***4.7***	***0.039***	***0.01(0.01-0.4)***
Open well	50	22	44	0.868	0.9(0.3-3.1)
Borehole	17	4	23.5	0.648	0.7(0.2-3.2)
Surface water	38	15	39.5	0.789	1.2(0.3-4.1)
Rain water	6	3	50	0.619	1.5(0.3-7.4)
Water vendors	9	3	33.3	Referent	Referent
**Type of drinking water storage container**					
Container with lid	70	8	11.4	0.733	0.9(0.4-1.9)
***Container without lid***	***59***	***31***	***52.5***	***0.004***	***1.9(1.1 - 3.7)***
Jerry cans	34	10	30.3	Referent	Referent
**Hand washing**					
***After using toilet***	***42***	***3***	***7.1***	***0.041***	***1.6(1.1-2.7)***
Before meals	59	27	45.8	0.719	0.9(0.5-1.7)
Before food preparation	62	19	30.6	Referent	Referent
**Use soap or hand sanitizers**					
Yes	62	16	25.8	0.439	0.8(0.4-1.4)
No	101	33	32.7	Referent	Referent
**Water treatment**					
Boiling	64	17	26.6	0.485	1.9(0.3-4.1)
Chemicals (chlorine)	52	18	34.6	0.868	1.4(0.3-4.1)
Filtration	3	0	0	ND	ND
Others	11	0	0	ND	ND
Stand and settle	33	14	42.4	Referent	Referent

No - Number; % - Percentage; OR - Odds ratio; CI - confidence interval

**Factors in multivariate analyses associated with infection with enteric pathogenic bacteria:** Table 5 summarizes the multivariate analysis of factors associated with infection with enteric pathogenic bacteria (ETEC, EPEC, EAEC, *Salmonella*, *Shigella*, and Vibrio). After adjusting for confounders; female children (OR 2.1, 95% CI 1.1-3.9), those who weighed 1 to 5 kilograms (OR 0.1, 95% CI 0.06-0.6) or 6 to 10 kilograms (OR 0.2, 95% CI 0.01-0.8), those who presented with watery stool (OR 0.4, 95% CI 0.2-0.9), and mucoid stool (OR 0.3, 95% CI 0.1-0.7), remained associated with enteric pathogenic bacterial infection.

**Table 5:** Adjusted factors associated with enteric pathogenic bacterial infection

		Enteric pathogenic bacterial infection			
Variables	Total			P - value	Multivariate
		No	%		OR (95% CI)
**Child gender**					
***Female***	***86***	***33***	***38.4***	***0.03***	***2.1(1.1 - 3.9)***
Male	77	16	20.8	Referent	Referent
**Weight (Kilograms)**					
***1 to 5***	***53***	***11***	***20.8***	***0.018***	***0.1(0.06-0.6)***
***6 to10***	***79***	***27***	***34.2***	***0.035***	***0.2(0.01-0.8)***
10 to15	29	9	31	0.065	0.1(0.01-1.1)
16 to 20	2	2	100	Referent	Referent
**Stool consistency**					
Watery	74	21	28.4	0.047	0.4(0.2-0.9)
***Mucoid***	***69***	***16***	***23.2***	***0.004***	***0.3(0.1 - 0.7)***
Bloody	2	0	0	ND	ND
Bloody and Watery	18	12	66.7	Referent	Referent
**Type of drinking water storage container**					
Container with lid	70	8	11.4	0.733	0.9(0.4-1.9)
***Container without lid***	***59***	***31***	***52.5***	***0.004***	***1.9(1.1 - 3.7)***
Jerry cans	34	10	30.3	Referent	Referent
**Hand washing**					
***After using toilet***	***42***	***3***	***7.1***	***0.041***	***1.6(1.1-2.7)***
Before meals	59	27	45.8	0.719	0.9(0.5-1.7)
Before food preparation	62	19	30.6	Referent	Referent

No - Number; % - Percentage; OR - Odds ratio; CI - confidence interval; ND - Not done

## Discussion

**Factors associated with diarrhea among the study participants:** bacteria that were frequently associated with diarrheal cases in children were Enterotoxigenic *Escherichia coli* (ETEC), Enteropathogenic *Escherichia coli* (EPEC), Enteroaggregative *Escherichia coli* (EAEC), *Salmonella* species, *Shigella* species, and Vibrio species. Forty-nine (49) children were infected with these enteric pathogenic bacteria and factors associated with the infection were evaluated.

**Age of the participants:** our finding that infants (below 1 year) were more likely to be infected with enteric pathogenic bacterial infection (OR 0.3, 95% CI 0.1-0.8) than the older ones conforms to a recent study done in Lusaka Zambia that implicated a high concentration of enterobacteriaceae infection on the ages of 0-12 months (61.3%) [15]. Weak maternal antibodies present during early stages of life may fairly protect the infants against bacterial infection but the host immunity heightens as the age of the child advances. A similar study in Kenya [16] and China [17] observed that the prevalence of diarrhea due to bacterial infection minimized as the age of the child increased, comporting our finding. Risk exposure to a myriad of enterobacteriaceae through complementary feeding may further result in frequent bacterial infections causing diarrhea and therefore plausible to assume that introducing food at 6 months elevated probable bacterial infection.

**Gender of the participants:** female participants (OR 1.8, 95% CI 1.1-3.4) were nearly twice likely to be infected with enteric pathogenic bacteria in this study which may be attributed to the difference in the immune system and other physiological factors between gender. Some studies have documented female children more prone to diarrheal-related bacterial infection but many factors may be attributed to this phenomenon. Our results are broadly similar to the work by Onyango and Angienda [16] in Kenya who reported that female between 6-36 months were twice likely to be infected by enterobacteriaceae than their male counterparts. In rural Sudan, an earlier study reported that female children less than 5 years were more at risk of diarrhea [18]. Our data output showed an association between gender with enteric pathogenic bacterial infection and female accounted for more than a double fold-frequency of isolated bacteria. Even though one gender may slightly be inflicted by bacterial pathogens, significance may not be strong enough to show a positive or negative association. For instance, even though male (54%) were more inflicted by diarrhea in a Nigerian teaching hospital, sex was not significantly related to the odds of having diarrhea [19]. Some authors, however, indicate an equal probability of diarrhea frequency between gender [20] while other studies in Kenya [21], Sudan [22], Tanzania [23] and India [24] contradict our finding indicating the male gender were more likely to be inflicted by enterobacteriaceae. Association between gender with enteric pathogenic bacterial infection among children less than 5 years is an area that requires further exploration due to various variables such as immunological factors, demographic features and environmental factors across the globe.

**Weight and Nutrition:** from our finding, the less heavy infants between 1-5 kilograms appeared to be protected (OR 0.2, 95% CI 0.04-0.9) against enteric pathogenic bacteria compared to heavier children who weighed 16 to 20 kilograms. It is possible that these infants were below 6 months and the majority were exclusively being breast-fed. Breastfeeding exclusively for 6 months or more appeared to have significantly reduced the risk of one or more episodes of Gastro-Intestinal Tract (GIT) infection [25] and less than 10 folds risk of death [26]. Diarrhea and malnutrition have a bi-directional relationship whereby the course of diarrhea worsens due to malnutrition [27]. Malnutrition alters vital metabolic processes such as reduced bile-acid synthesis, digestive enzymes, motility and shift of gut flora elevating probability of enteric infections causing diarrhea. Vibrio cholera infection leads to loss of 1 litre of water per hour resulting in severe hypotensive shock and high case fatality rates [28]. Repeated frequencies of enteric infections and diarrhea results in poor nutrient absorption. Specifically, from our study, nutrition status was not associated with enteric pathogenic bacterial infection and this finding runs consistently with an observation from an earlier study in Bangladesh that failed to demonstrate this relationship among pre-school children [29]. Our finding, however, is at odds with a study that demonstrated the existence of a relation between malnutrition and diarrhea [30]. An explanation of our finding may be based on the nutritional supplements that were prescribed in the Maternal and Child Health (MCH) booklet. It is likely that the majority of the children who were attending postnatal clinics and had nutrition problems were on supplements to correct malnutrition and hence were less associated with diarrhea.

**Breastfeeding:** breastfeeding was found associated with enteric pathogenic bacterial infection. Those fed exclusively on breast milk (OR 0.3, 95% CI 0.09-0.9) were less likely to be infected with these enteric pathogenic bacteria compared to those who were currently not breastfeeding. Passive immunity from transplacental and breast milk antibodies protects the infant within the first six weeks [1]. Children who failed to breastfeed exclusively (first 6 months) were more than 3 folds at risk of contracting diarrhea in Imenti, Meru [31] and more than seven and a half times among refugee children [32]. Evidence indicates that breast milk contains vital components such as antioxidants, antibodies, hormones and nutrients which are difficult to incorporate in other fluids and thus offers the greatest protection against enteric infections. Nearly all participants stated to breastfeed or having breastfed infants below 6 months and can be said to have met local credibility. This study affirms that breast milk offers the greatest protection against infectious agents such as enterobacteriaceae especially during the first 6 months of life.

**Fever:** fever was an apparent clinical symptom but unfortunately not associated with enteric pathogenic bacterial infections. Nevertheless, 46 out of the 49 children infected with enteric pathogenic bacterial isolates recorded a body temperature beyond 37.5 degrees Celsius and documented as fever. In line with the finding in this context, studies in Nairobi [33], India [24] and China [17] have observed that fever was a prevalent clinical symptom among children less than 5 years with diarrhea. It can be deciphered that infection by enteric gram-negative bacteria may have triggered the frequent fevers but a deficiency of our analysis could not determine the number of bacterial isolates per each child affected. We can deduce our finding from the complex fever cascade mechanism where the Lipopolysaccharide Binding Proteins (LBP) binds to the gram-negative lipopolysaccharide (LPS) forming LBP-LPS complex that binds to macrophages and activates the release of endogenous cytokines (interleukin-1, interleukin-6, and tumor necrosis-α) [34]. Prostaglandin E2 is in turn triggered by these pyrogens thus stimulate the hypothalamus generating a systemic response causing a rise in the temperature.

**Stool appearance:** children who presented with watery stool (OR 0.4, 95% CI 0.2-0.9) or mucoid stool (OR 0.3, 95% CI 0.2-0.7) remained associated with enteric pathogenic bacterial infection but were less likely to be infected with these bacteria compared to children who presented with bloody and watery stool. Dysentery is defined by the presence of blood in the loose stool. This context provided strong evidence of a relationship between bloody stools and the enteric pathogenic bacterial infection which runs in harmony with finding reported in Kenya [33]. Invasive enteric bacterial pathogens in most cases cause bloody diarrhea that can be either watery or mucoid with recent evidence recommending culturing of all bloodstained stools for isolation of E. coli O517: H7.

**Water sources and sourcing:** the type of water source was found associated with enteric pathogenic bacterial infection. Those who sourced water by piping (OR 0.01, 95% CI 0.01-0.4) were less likely to be infected with enteric pathogenic bacteria than those who sourced water from water vendors. Only 2(4.7%) participants who received water by piping were infected by enteric pathogenic bacteria. Water supplied by piping was treated by Murang'a Water and Sanitation Company (MUWASCO) before pumping within the urban and some rural areas within the households hence the low infection frequency of the study participants who received water by piping compared to other modes. Contamination of water at any level before use is possible but treating it at the source minimizes chances of contracting diarrhea [35]. Protecting drinking water with a lid was less associated with bacterial infection than uncovered water in jerry-cans. Covering water prevents contamination from disease-causing microorganisms within the environment [36]. Drawing and storage of drinking water from same sources where water for other chores such as bathing was not associated with enteric pathogenic bacterial infection rhyming with finding reported in a different study [16]. Majority of the guardians stated that they always or at times stored drinking water separately from that of other domestic purposes in the current study. Disposal of sluice water was not statistically significant nor associated with enteric pathogenic bacterial infection.

**Mode of water storage:** finding in this study showed that storing water without a lid (OR 1.9, 95% CI 1.1-3.7) was associated with enteric pathogenic bacterial infection compared to storing water using jerry cans. A study in Tanzania chimes with these finding and reported that storing water without lids was attributed to an increased risk of contracting diarrhea among children below 5 years [37]. Despite the high numbers of study participants sourcing water from surface sources and open wells, the majority (42.9%) used covered containers and nearly all study participants reported to have used effective treatment strategies such as chlorination and boiling that are known to minimize bacterial agents attributed with diarrhea.

**Hand washing and water treatment:** washing hands after toilet use (OR 1.6, 95% CI 1.1-2.7) was more likely to lead to infection with enteric pathogenic bacteria compared to those who washed hands before meal preparation. Most enteric bacteria are destroyed and killed by heat during cooking which correlates with minimized numbers of micro-organisms on the background while fecal contamination may have numerous and a wide range of enteric pathogenic bacteria requiring a minimal dose to cause an infection. Our outcome may also be attributed by the fact that nearly all respondents in this study (94.5%) purported to have used water treatment methods, a wide difference from what was observed from a study in Tanzania (49.7%) [36]. Treated water, both for drinking and cooking, killed most pathogenic microbes making it safe for human consumption and reduces bacterial acquisition as observed in our study. For instance, following treatment with 16ml of 1% chlorine, E. coli coliforms in untreated river water for 20 litres jerry achieved adequate disinfection [10]. Moreover, nearly three quarter (3/4) of the study participants stated that they never used soap or any sanitizer to wash their hands and were twice infected by enteric pathogenic bacteria than those who used soap or sanitizer to wash their hands. Independent factors associated to having diarrhea among the children included caregivers not using soap to hand-wash in a different study [36]. Using water alone to hand wash has been found to be a less effective intervention and studies have pointed out that the use of soap prolongs hand washing period as well as break grease and dirt harboring pathogens thereby minimizing diarrhea risk by 42-47% [38]. Lack of soap use or sanitizer among the majority of the study participants might have been an attributable factor that correlated with bacterial agents causing diarrhea, especially after toilet use.

## Conclusion

Sanitation, hygiene, nutrition and clinical factors were associated with enteric bacterial infection causing diarrhea among children below five years in Murang'a County in Kenya. Emphasis on public health promotion programs would be a good start-off in addressing deficiencies in preventive strategies aimed at minimizing enteric diarrheal illnesses among children below five years in Murang'a County.

### What is known about this topic

- Poor hygiene and sanitation factors are associated with diarrhea;
- Enteric bacterial infections are a major cause of diarrhea among children below five years.

### What this study adds

- Major enteric pathogenic bacteria (*Salmonella*, *Shigella*, ETEC, EPEC and, EAEC) are associated with diarrhea among children below five years in Murang'a County in Kenya;
- Hygiene, sanitation, gastrointestinal and clinical factors are major drivers of an enteric bacterial infection causing diarrhea among children below five years in Murang'a County in Kenya.

## Competing interests

The author declares no competing interests.
